# CD14^+^CD16^−^ monocytes are the main precursors of osteoclasts in rheumatoid arthritis via expressing Tyro3TK

**DOI:** 10.1186/s13075-020-02308-7

**Published:** 2020-09-21

**Authors:** Jimeng Xue, Liling Xu, Huaqun Zhu, Mingxin Bai, Xin Li, Zhen Zhao, Hua Zhong, Gong Cheng, Xue Li, Fanlei Hu, Yin Su

**Affiliations:** 1 Department of Rheumatology and Immunology, Peking University People’s Hospital, 11 Xizhimen South Street, Beijing, 100044 China; 2 Beijing Key Laboratory for Rheumatism Mechanism and Immune Diagnosis (BZ0135), Beijing, China; 3 Peking-Tsinghua Center for Life Sciences, Peking University, Beijing, China; 4 State Key Laboratory of Natural and Biomimetic Drugs, School of Pharmaceutical Sciences, Peking University, Beijing, China

**Keywords:** Rheumatoid arthritis, Monocyte subsets, Osteoclast, Tyro3TK

## Abstract

**Background:**

Monocytes as precursors of osteoclasts in rheumatoid arthritis (RA) are well demonstrated, while monocyte subsets in osteoclast formation are still controversial. Tyro3 tyrosine kinase (Tyro3TK) is a member of the receptor tyrosine kinase family involved in immune homeostasis, the role of which in osteoclast differentiation was reported recently. This study aimed to compare the osteoclastic capacity of CD14^+^CD16^+^ and CD14^+^CD16^−^ monocytes in RA and determine the potential involvement of Tyro3TK in their osteoclastogenesis.

**Methods:**

Osteoclasts were induced from CD14^+^CD16^+^ and CD14^+^CD16^−^ monocyte subsets isolated from healthy control (HC) and RA patients in vitro and evaluated by tartrate-resistant acid phosphatase (TRAP) staining. Then, the expression of Tyro3TK on CD14^+^CD16^+^ and CD14^+^CD16^−^ monocyte subsets in the peripheral blood of RA, osteoarthritis (OA) patients, and HC were evaluated by flow cytometry and qPCR, and their correlation with RA patient clinical and immunological features was analyzed. The role of Tyro3TK in CD14^+^CD16^−^ monocyte-mediated osteoclastogenesis was further investigated by osteoclast differentiation assay with Tyro3TK blockade.

**Results:**

The results revealed that CD14^+^CD16^−^ monocytes were the primary source of osteoclasts. Compared with HC and OA patients, the expression of Tyro3TK on CD14^+^CD16^−^ monocytes in RA patients was significantly upregulated and positively correlated with the disease manifestations, such as IgM level, tender joint count, and the disease activity score. Moreover, anti-Tyro3TK antibody could inhibit Gas6-mediated osteoclast differentiation from CD14^+^CD16^−^ monocytes in a dose-dependent manner.

**Conclusions:**

These findings indicate that elevated Tyro3TK on CD14^+^CD16^−^ monocytes serves as a critical signal for osteoclast differentiation in RA.

## Background

Rheumatoid arthritis (RA) is one of the most common chronic systemic inflammatory rheumatic disease hallmarked by synovitis, aggressive lesions of the articular cartilage and bone, which leads to irreversible joint deformity and loss of function [1–3]. Bone erosion is the main pathological change in RA, which can even be observed in more than 45% of RA patients at an early stage [4]. It has been proved that excessive activation of local osteoclasts is involved in focal bone erosion in RA [5]. Osteoclasts are multinucleated cells which derived from the monocyte/macrophage lineage, especially from CD14^+^ monocytes [6].

Monocytes are plastic cells that can differentiate into macrophages, dendritic cells, and osteoclasts, which can accumulate in the blood and continuously migrate to inflammatory joints. Expanded monocytes in RA patients can lead to chronic joint inflammation and bone destruction [7]. Recently, based on differential surface expression of CD14 and CD16, human monocytes could be subdivided into two major subsets: CD14^+^CD16^+^ and CD14^+^CD16^−^ monocytes, accounting for 5–10% and 90–95% of monocytes in healthy individuals, respectively [8].

However, the role of CD14^+^CD16^+^ and CD14^+^CD16^−^ monocytes in osteoclast formation is still controversial. Bolzoni et al. demonstrated that bone marrow CD14^+^CD16^+^ monocytes from patients with multiple myeloma tended to differentiate into osteoclasts more remarkably than CD14^+^CD16^−^ monocytes [9]. Chiu et al. also suggested that CD16^+^ monocytes from psoriatic arthritis patients were more prone to differentiate to osteoclasts [10]. In contrast, several studies illustrated that the osteoclasts were mainly derived from the CD14^+^CD16^−^ monocytes in healthy donors [10–12]. Komano et al. further demonstrated that CD14^+^CD16^−^ monocytes rather than CD14^+^CD16^+^ monocytes were the circulating osteoclast precursors in RA recently [11]. The different microenvironments of diseases would shape the phenotype of monocyte subsets and influence their capacity of osteoclast differentiation. In particular, studies have shown that multiple myeloma cells could profoundly modify the immune functions of the bone marrow cells as well as the bone marrow microenvironment [13, 14]. All these suggest that peripheral blood monocyte subsets may be directly involved in exacerbated osteoclast formation in RA. However, which monocyte subsets are the major sources of osteoclasts remains elusive.

Tyro3 tyrosine kinase (Tyro3TK) is one of the family members of TAM (Tyro3TK, AxlTK, MerTK) receptor tyrosine kinases (RTKs) [15], which could be expressed on the plasma membrane of a variety of cells, such as monocytes/macrophages, dendritic cells, NK cells, and nerve cells [16]. Tyro3TK could regulate the clearance of apoptotic cells, cytokine production, cell proliferation, thrombus formation, and hematopoiesis by binding to its ligand growth arrest-specific protein 6 (Gas6) and protein S (ProS1) [17, 18]. It was reported that Gas6 is expressed in RA synovium tissue and fluid and plays a role in RA synovium endothelial cell survival [19]. Furthermore, the expression of Gas6 appears to be stimulated by an inflammatory response, since elevated serum Gas6 levels were shown in sepsis and other systemic inflammation [20].

In 1998, Nakamura et al. firstly identified that Tyro3TK could be expressed in multinucleated osteoclasts, and the bone resorption activity of mature osteoclasts can be enhanced when binding with the ligand Gas6. However, Tyro3TK did not affect the differentiation of osteoclasts from bone marrow cells [21]. Katagiri et al. also found that Tyro3TK can be detected in mature osteoclasts while they showed that Gas6 demonstrated no apparent effect on osteoclast formation in mouse osteoclast progenitor cells [22]. Kawaguchi et al. found that Tyro3TK can only be detected in mouse mature osteoclasts among bone cells, while Gas6 is widely expressed in bone cells, stimulating the function of osteoclasts [23]. Recently, Ruiz-Heiland et al. illustrated that Tyro3TK-deficient mice showed an increased bone mass and impaired osteoclast differentiation in the arthritis model, suggesting the involvement of Tyro3TK in the differentiation and functional maturation of osteoclasts [24]. All these indicated that Tyro3TK might play a critical role in bone destruction in inflammatory arthritis. Despite these findings, the expression and osteogenic function of Tyro3TK on monocyte subsets in RA remain largely unknown.

In this study, we compared the osteoclastic capacity of CD14^+^CD16^+^ and CD14^+^CD16^−^ monocytes in RA and determined the expression levels as well as the potential involvement of Tyro3TK in their osteoclastogenesis, aiming to further understand the mechanism of RA bone destruction.

## Methods

### Patients and controls

Fifty-seven patients with RA (Table 1), 28 osteoarthritis (OA) patients, and 49 age- and sex-matched healthy controls (HC) were enrolled in this study. All the patients met the 2010 American College of Rheumatology (ACR) revised criteria for RA [25] and 1986 ACR criteria for OA [26]. The study was approved by the Institutional Medical Ethics Review Board of Peking University People’s Hospital. Moreover, all participants provided informed consent.

**Table 1:** Demographic and clinical characteristics of RA patients

Characteristics	RA (***n*** = 57)
Age, mean (range), years	59 (23–83)
Sex, no, female/male	44/13
Duration, mean (range), years	14.7 (0.25–58)
SJC, median (range) of 28 joints	2 (0–28)
TJC, median (range) of 28 joints	6 (0–28)
RF, mean (range), IU/ml	319.2 (20–5660)
Anti-CCP antibody, mean (range), IU/ml	168.1 (2.72–311)
ESR, mean (range), mm/h	47.4 (6–115)
CRP, mean (range), mg/l	31.5 (0.27–124)
DAS28-ESR, mean (range)	6.42 (1.25–11.94)

*RA* rheumatoid arthritis, *SJC* swollen joint count, *TJC* tender joint count, *RF* rheumatoid factor, *Anti-CCP antibody* anti-cyclic citrullinated peptide antibody, *ESR* erythrocyte sedimentation rate, *CRP* C-reactive protein, *DAS28* Disease Activity Score 28

### Clinical and laboratory indices of RA

The following data of patients with RA were recorded: gender, age, duration, swollen joint count (SJC), tender joint count (TJC), and laboratory parameters including white blood cells (WBC), red blood cells (RBC), hemoglobin (Hb), platelets (PLT), immunoglobulin (Ig) A, IgG, IgM, anti-cyclic citrullinated peptide antibody (anti-CCP antibody), erythrocyte sedimentation rate (ESR), and C-reactive protein (CRP). Disease activity scores were calculated using the 28-joint Disease Activity Score-erythrocyte sedimentation rate (DAS28-ESR) in patients with RA. DAS28-ESR > 5.1 was considered a high disease activity according to the recommendations from the European League Against Rheumatism (EULAR).

### Antibodies and reagents

Recombinant human macrophage colony-stimulating factor (rhM-CSF) (Cat# 300-25) was obtained from PerproTech GmbH (Rocky Hill, CT). Recombinant human RANKL (rhRANKL) (Cat# 390-TN), recombinant human Gas6 (rhGas6) (Cat# 885-GSB), human anti-Tyro3TK antibody (Cat# MAB859, Clone# 96201) proved to demonstrate blocking activity [27], human Tyro3TK PE-conjugated antibody (Cat# FAB859P), and mouse IgG2b PE-conjugated antibody (Cat# IC0041P) were purchased from R&D Systems (Minneapolis, MN). Human TruStain FcX™ (Fc Receptor Blocking Solution) (Cat# 422302) was purchased from BioLegend (San Diego, CA). Human CD14 FITC-conjugated antibody (Cat# 11-0141-81) and human CD16 APC-conjugated antibody (Cat# 17-0168-42) were purchased from eBioscience (San Diego, CA). The Leukocyte Acid Phosphatase Kit (Cat# 387A) was purchased from Sigma-Aldrich (St. Louis, MO). α-Minimum Essential Medium (α-MEM) (Cat# C11965500BT), 1% penicillin/streptomycin, and fetal bovine serum were purchased from Invitrogen (Carlsbad, CA).

### Flow cytometry analysis and sorting

Peripheral blood mononuclear cells (PBMCs) were isolated from fresh EDTA blood samples using Ficoll density gradient centrifugation. Before staining with antibodies, single-cell suspensions were incubated with human Fc Receptor Blocking Solution for 10 min at room temperature to block the FcR-involved unwanted staining without interfering with antibody-mediated specific staining.

To detect the expression of Tyro3TK on CD14^+^CD16^+^ and CD14^+^CD16^−^ monocytes, cells were stained with CD14 FITC-conjugated antibody, CD16 APC-conjugated antibody, and Tyro3TK PE-conjugated antibody. Corresponding negative isotype and fluorochrome-matched control (FMO) staining were also performed. The cells were then analyzed on FACS Aria II.

For CD14^+^CD16^+^ and CD14^+^CD16^−^ monocyte sorting, cells were stained with CD14 FITC-conjugated antibody and CD16 APC-conjugated antibody. Then, the stained cells were sorted with FACS Aria II. The purified CD14^+^CD16^+^ and CD14^+^CD16^−^ monocytes were further analyzed after sorting, the purity of which used for experiments was ~ 90%.

### qPCR analysis of Tyro3TK expression

Total RNA was isolated from purified CD14^+^CD16^−^ monocytes using the RNeasy mini kit (Qiagen, Hilden) then reverse transcribed into the oligo (dT)-primed cDNA by Revert Aid First Strand kit (Fermentas, Glen Burnie, MD). Real-time quantitative PCR (qPCR) was performed to analyze the expression of Tyro3TK mRNA in CD14^+^CD16^−^ monocytes from RA patients and HC according to the manufacturer’s instructions. The sequences of the primers used in this study were as follows: the forward GAPDH primer was 5′-AAGGTGAAGGTCGGAGTCAA-3′, the reverse GAPDH primer was 5′-AATGAAGGGGTCATTGATGG-3′, the forward Tyro3TK primer was 5′-CAGCCGGTGAAGCTCAACT-3′, and the reverse Tyro3TK primer was 5′-TGGCACACCTTCTACCGTGA-3′.

### In vitro osteoclast differentiation

CD14^+^CD16^+^ and CD14^+^CD16^−^ monocytes from freshly isolated PBMCs were purified by FACS sorting. Then, the cells were cultivated 17 days separately in 96-well plates (5 × 10^4^ cells/200 μl per well) in α-MEM with 1% PenStrep, 10% heat-inactivated fetal bovine serum, 30 ng/ml rhM-CSF, and 50 ng/ml rhRANKL. Different concentrations of rhGas6 and/or human anti-Tyro3TK antibody were added as indicated. The medium was changed with fresh medium every 6 days. Osteoclast differentiation was evaluated by staining cells for TRAP using a Leukocyte Acid Phosphatase kit (Sigma-Aldrich) according to the manufacturer’s instructions. TRAP-positive multinucleated cells were counted by an inverted fluorescence microscope (Olympus IX71-141, Tokyo, Japan).

### Statistical analysis

All data were analyzed on the statistical software program SPSS 24.0 for Windows (SPSS, Chicago, IL). Differences between the groups were evaluated by Student’s *t* test, non-parametric Mann-Whitney *U* test, one-way ANOVA test, Kruskal-Wallis *H* test, and Spearman’s correlation test. *P* value less than 0.05 was considered statistically significant (*P < 0.05, ** *P* < 0.01, *** *P* < 0.001; ns, not significant).

## Results

### CD14^+^CD16^−^ monocytes are the main precursors of osteoclasts in RA

**Fig. 1:**
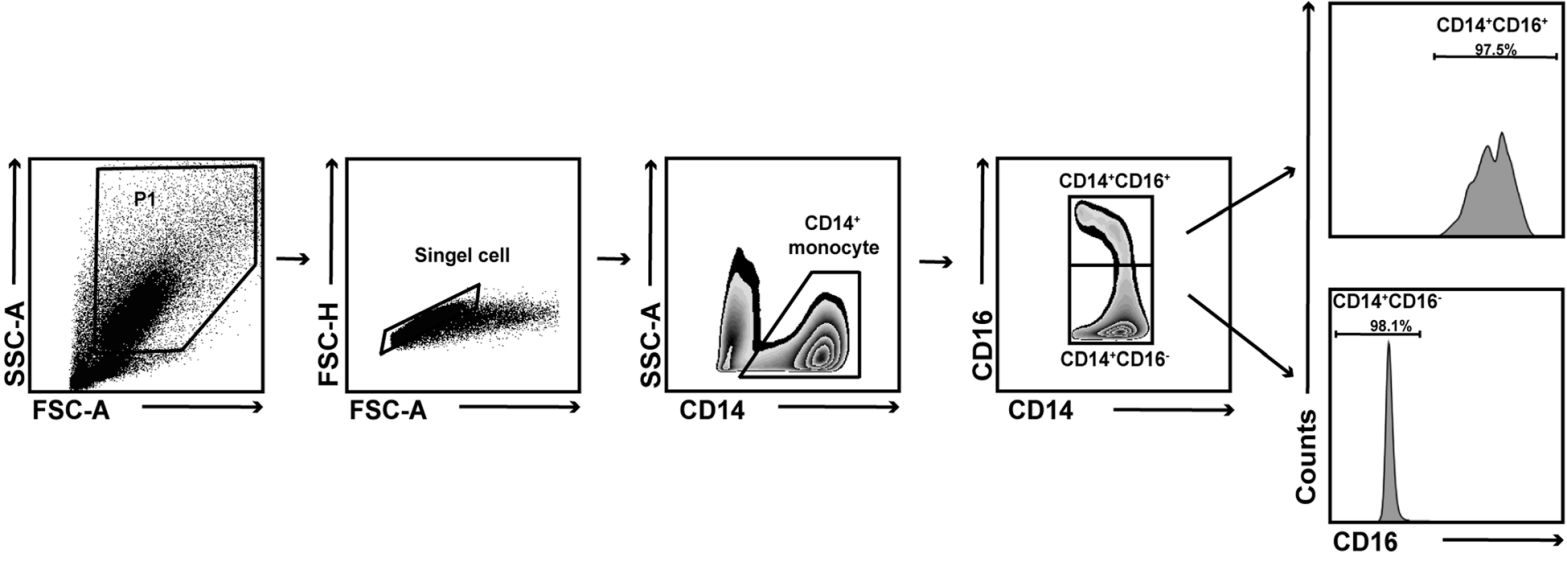
Gating strategy for flow cytometry sorting of human CD14^+^CD16^+^ and CD14^+^CD16^−^ monocytes. Peripheral blood mononuclear cells from RA and HC were stained with FITC-conjugated anti-CD14 antibody and APC-conjugated anti-CD16 antibody. CD14^+^CD16^+^ and CD14^+^CD16^−^ monocytes were analyzed and sorted by flow cytometric; the purity of sorted CD14^+^CD16^+^ and CD14^+^CD16^−^ monocytes used for experiments was ~ 90%

**Fig. 2:**
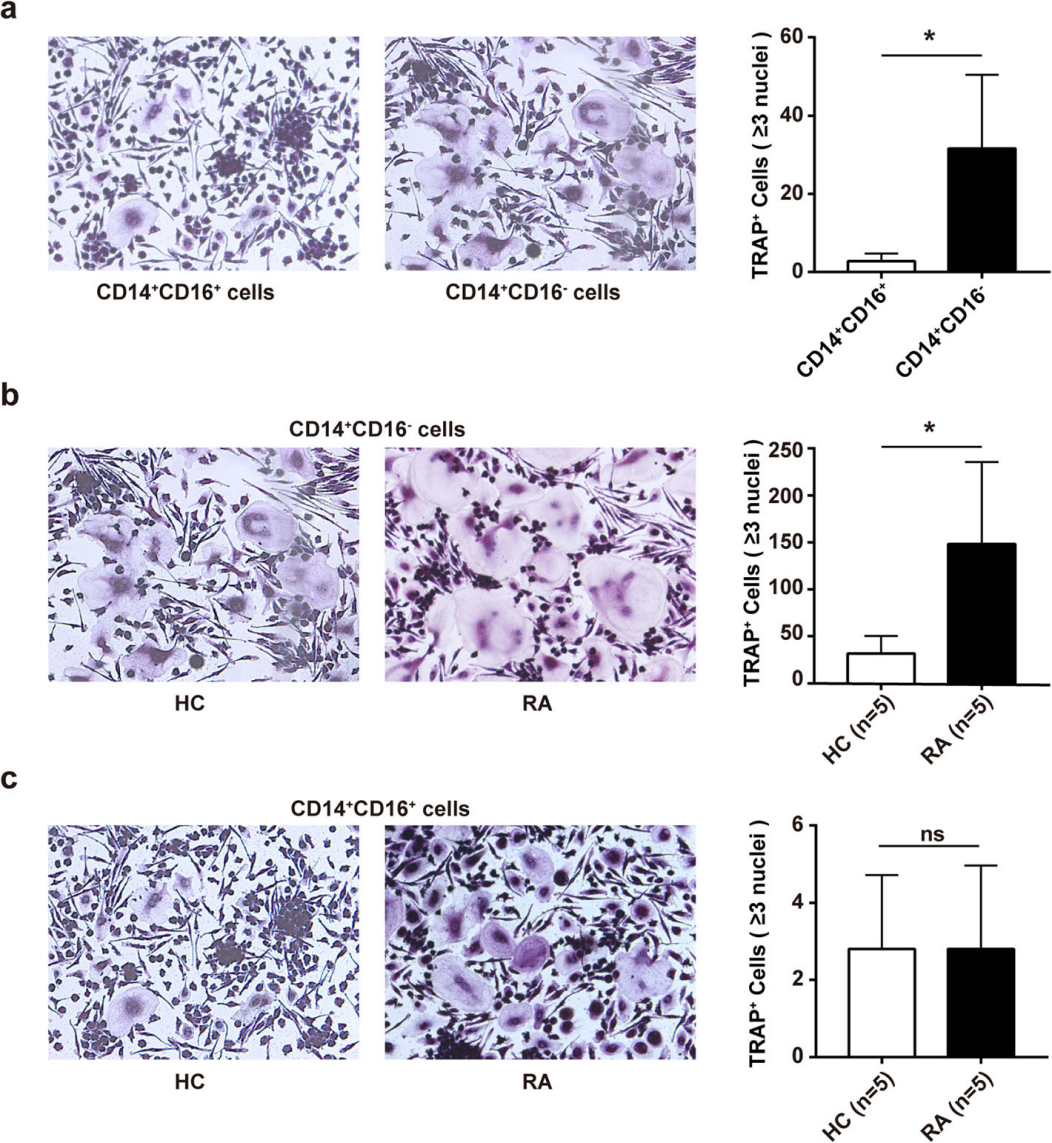
CD14^+^CD16^−^ monocytes are the main osteoclast precursors in RA. Purified CD14^+^CD16^+^ and CD14^+^CD16^−^ monocytes from RA (*n* = 5) and HC (*n* = 5) were cultured with rhM-CSF (30 ng/ml) and rhRANKL (50 ng/ml) for osteoclast differentiation. The cells were detected for tartrate-resistant acid phosphatase (TRAP) staining on day 17, and the TRAP-positive multinuclear cells were osteoclasts. The representative charts and the statistical results were shown. **a** CD14^+^CD16^+^ versus CD14^+^CD16^−^ monocytes in HC (*P = 0.026). **b** RA versus HC for CD14^+^CD16^−^ monocytes (*P = 0.019). **c** RA versus HC for CD14^+^CD16^+^ monocytes. *P < 0.05; ns, not significant (Student’s *t* test, **a**−**c**)

To reveal which monocyte subset plays a significant role in osteoclast formation in RA, we performed osteoclast differentiation assay with monocyte subpopulation in vitro. CD14^+^CD16^+^ and CD14^+^CD16^−^ monocytes were isolated from 5 HC and 5 RA patients by FACS sorting, respectively, the purity of which was confirmed by FACS (Fig. 1). Then osteoclast differentiation and TRAP staining were performed. Interestingly, the results showed that the number of TRAP-positive osteoclasts differentiated from CD14^+^CD16^−^ monocytes were much more than that from CD14^+^CD16^+^ monocytes in HC (Fig. 2a). Moreover, CD14^+^CD16^−^ monocytes demonstrated upregulated capacity of osteoclast differentiation in RA patients (Fig. 2b). However, there was no distinct difference for CD14^+^CD16^+^ monocytes between RA patients and HC (Fig. 2c).

### Expression of Tyro3TK is enriched on CD14^+^CD16^−^ monocytes and upregulated in RA patients

**Fig. 3:**
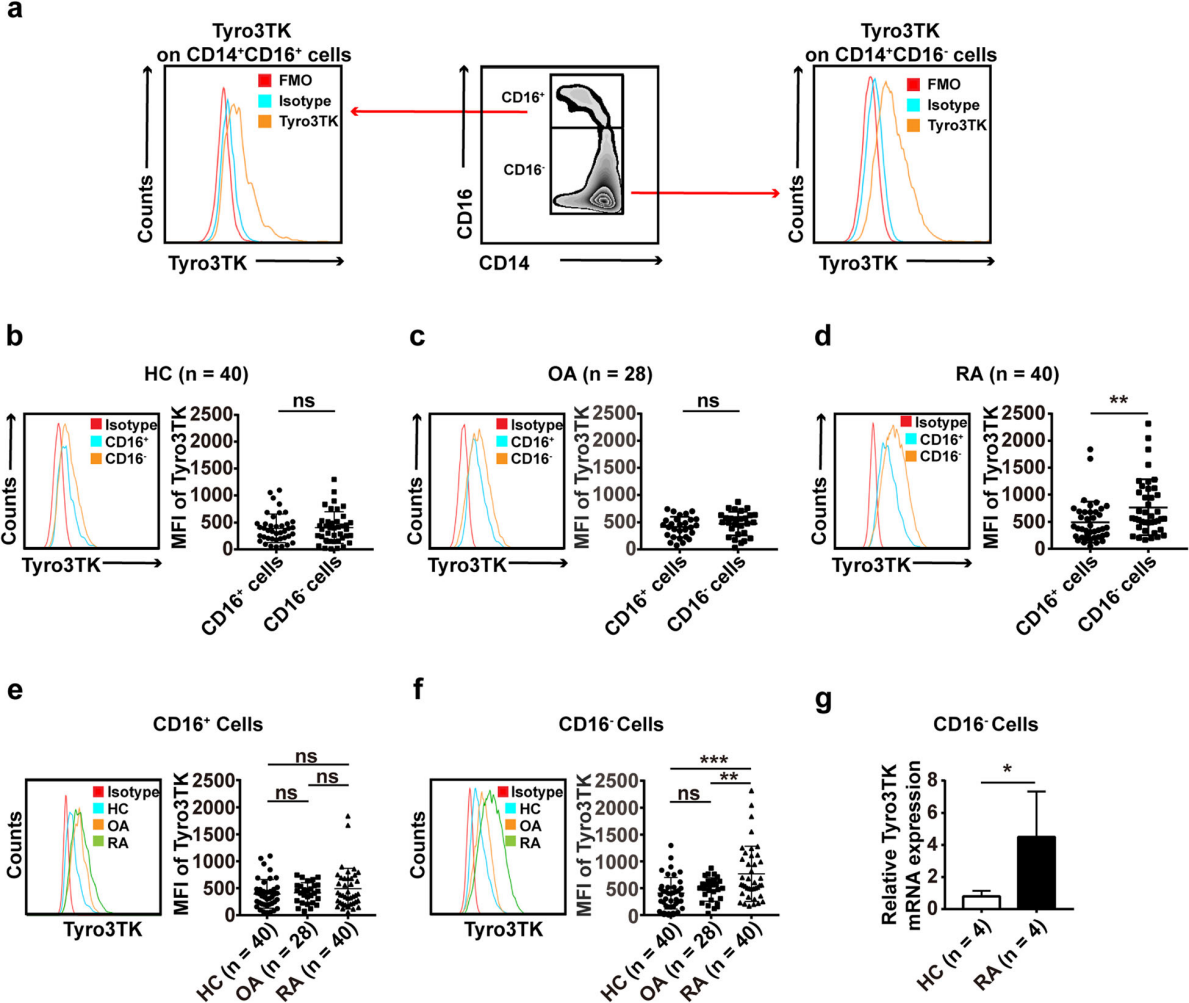
The expression of Tyro3TK on CD14^+^CD16^−^ monocytes is increased in RA. **a** Gating strategy for identifying the expression of Tyro3TK on CD14^+^CD16^+^ and CD14^+^CD16^−^ monocytes. Accordingly, the expression of Tyro3TK on CD14^+^CD16^+^ and CD14^+^CD16^−^ monocytes in HC (*n* = 40) (**b**), OA (*n* = 28) (**c**), and RA patients (*n* = 40, **P = 0.008) (**d**) were analyzed and presented as the mean fluorescence intensity (MFI). **e** The expression of Tyro3TK on CD14^+^CD16^+^ monocytes were compared between HC, OA, and RA patients. **f** The expression of Tyro3TK on CD14^+^CD16^−^ monocytes were compared between HC, OA, and RA patients (**P = 0.004, ***P < 0.001). **g** Flow cytometry-sorted CD14^+^CD16^−^ monocytes from RA (*n* = 4) and HC (*n* = 4) were set to detect the mRNA expression of Tyro3TK by qPCR (*P = 0.029). *P < 0.05, **P < 0.01, ***P < 0.001; ns, not significant (Mann-Whitney *U* test, **b**, **d**, and **g**; Student’s *t* test, **c**; Kruskal-Wallis test followed by Dunn’s post-test for multiple comparisons, **e**–**f**)

Then, we tried to reveal the effects of Tyro3TK on monocyte subset-mediated osteoclast differentiation. The expression of Tyro3TK on monocyte subsets in RA patients, OA patients, and HC were first analyzed and presented as mean fluorescence intensity (MFI). The gating strategy was demonstrated in Fig. 3a. We identified that there was no apparent difference in the expression of Tyro3TK on CD14^+^CD16^+^ and CD14^+^CD16^−^ monocytes in HC and OA (Fig. 3b, c). Interestingly, the expression of Tyro3TK on CD14^+^CD16^−^ monocytes in patients with RA was significantly higher than that of CD14^+^CD16^+^ monocytes (Fig. 3d). Moreover, the expression of Tyro3TK on CD14^+^CD16^−^ monocytes was significantly increased in RA patients as compared with OA patients and HC. However, no significant difference was found for Tyro3TK expression on CD14^+^CD16^+^ monocytes between RA patients, OA patients, and HC (Fig. 3e, f). To further confirm our findings, we also detected the mRNA expression of Tyro3TK by qPCR. As shown in Fig. 3g, the results revealed that compared with HC, RA patient CD14^+^CD16^−^ monocytes expressed significantly higher levels of Tyro3TK transcripts.

### Tyro3TK on CD14^+^CD16^−^ monocytes are associated with RA patient clinical and immunological features

**Fig. 4:**
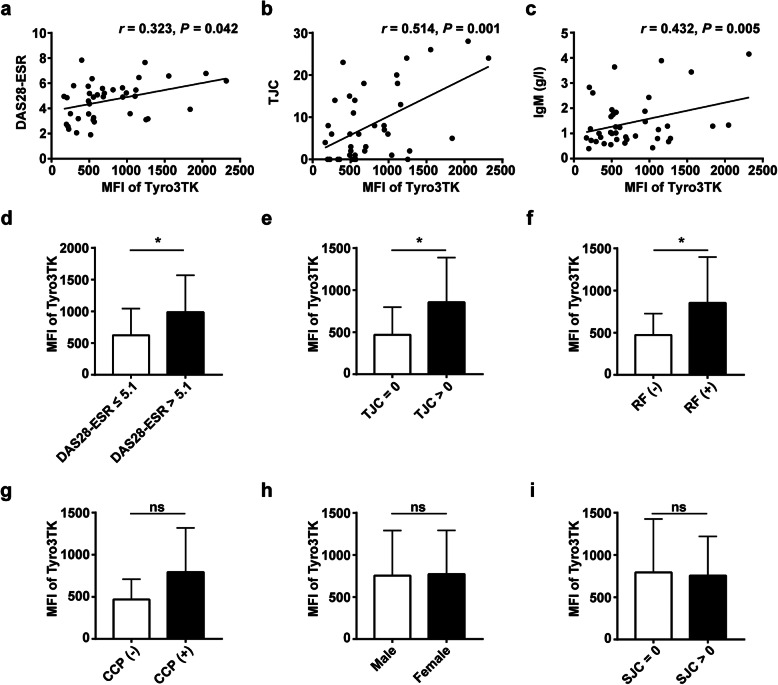
Correlation analysis of Tyro3TK on CD14^+^CD16^−^ monocytes with RA patient clinical manifestations. The associations of Tyro3TK on CD14^+^CD16^−^ monocytes with RA patient DAS28-ESR (*r* = 0.323, *P = 0.042) (**a**), tender joint counts (TJC) (*r* = 0.514, **P = 0.001) (**b**), and IgM (*r* = 0.432, **P = 0.005) (**c**) were analyzed. The expression of Tyro3TK on CD14^+^CD16^−^ monocytes were also compared between the different RA patient groups: **d** RA with high disease activity (DAS28-ESR > 5.1) and non-high disease activity (DAS28-ESR ≤ 5.1) (*P = 0.034), **e** RA with and without tender joints (*P = 0.031), **f** rheumatoid factor (RF) positive and negative RA (*P = 0.024), **g** anti-CCP antibody positive and negative RA, **h** male and female RA, and **i** RA with and without swollen joints. *P < 0.05, **P < 0.01; ns, not significant (Spearman’s rank correlation test, **a**–**c**; Mann-Whitney *U* test, **d**–**i**)

Then, we analyzed the correlation of Tyro3TK on CD14^+^CD16^+^ and CD14^+^CD16^−^ monocytes with RA patient clinical and immunological features, respectively. The results revealed substantial associations (Table 2). Notably, the levels of Tyro3TK on CD14^+^CD16^−^ monocytes were found to be positively correlated with DAS28-ESR, TJC, and serum IgM (Fig. 4a–c). Detailed analyses showed that RA patients with high disease activity (DAS28-ESR > 5.1) showed higher levels of Tyro3TK on CD14^+^CD16^−^ monocytes (Fig. 4d). Similar results were also seen in RA patients with tender joints and RF positivity (Fig. 4e, f). However, no apparent association was found between the levels of Tyro3TK on CD14^+^CD16^−^ monocytes and RA patient’s gender, anti-CCP, or swollen joints (Fig. 4g–i).

**Table 2:** Correlation of Tyro3TK expression on monocyte subsets with RA patient clinical and immunological features

Features	Tyro3TK on CD14^+^CD16^+^ monocytes		Tyro3TK on CD14^+^CD16^−^ monocytes	
	*r*	*P*	*r*	*P*
Age	− 0.005	0.974	0.071	0.664
Duration	− 0.077	0.637	− 0.071	0.664
WBC	0.15	0.357	0.097	0.554
RBC	0.03	0.853	0.041	0.803
Hb	− 0.023	0.889	0.035	0.831
PLT	0.092	0.572	− 0.012	0.941
ESR	0.088	0.59	0.104	0.522
CRP	0.118	0.469	0.072	0.659
IgA	0.222	0.169	0.196	0.225
IgG	0.106	0.52	0.099	0.547
**IgM**	0.348^*^	**0.028**	0.432**	**0.005**
RF	0.136	0.402	0.108	0.509
Anti-CCP antibody	0.192	0.243	0.172	0.295
**TJC**	0.459**	**0.003**	0.514**	**0.001**
SJC	0.054	0.741	0.043	0.793
**DAS28-ESR**	0.28	0.08	0.323^*^	**0.042**

The date was analyzed by Spearman’s correlation coefficient test

*WBC* white blood cells, *RBC* red blood cells, *Hb* hemoglobin, *PLT* platelets, *ESR* erythrocyte sedimentation rate, *CRP* C-reactive protein, *IgA/G/M* immunoglobulin A/G/M, *RF* rheumatoid factor, *Anti-CCP antibody* anti-cyclic citrullinated peptide antibody, *TJC* tender joint count, *SJC* swollen joint count, *DAS* Disease Activity Score

*P < 0.05

**P < 0.01

### Upregulated Tyro3TK on CD14^+^CD16^−^ monocytes promotes their osteoclast differentiation in RA

**Fig. 5:**
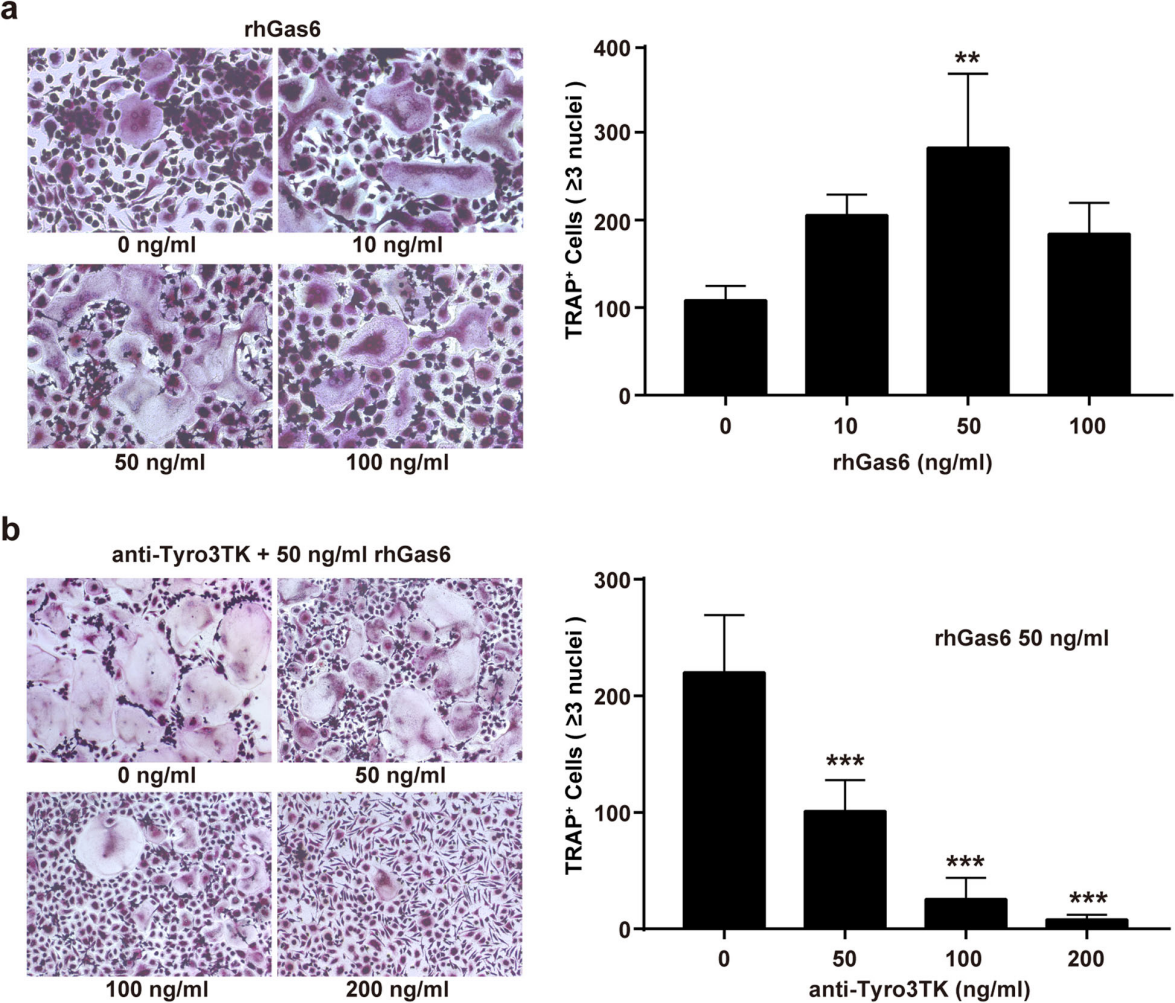
Tyro3TK promotes CD14^+^CD16^−^ monocyte-mediated osteoclastogenesis in RA. Purified CD14^+^CD16^−^ monocytes from RA patients (*n* = 8) were cultured with rhM-CSF (30 ng/ml) and rhRANKL (50 ng/ml) under different conditions for osteoclast differentiation. Seventeen days later, the cells were harvested for TRAP staining. The representative charts and the statistical results were shown. **a** Different concentrations of rhGas6 (0 ng/ml, 10 ng/ml, 50 ng/ml, and 100 ng/ml) were supplemented for osteoclast differentiation (*n* = 3 per group, **P = 0.006). **b** Different concentrations of anti-Tyro3TK antibody (0 ng/ml, 50 ng/ml, 100 ng/ml, and 200 ng/ml), and 50 ng/ml rhGas6 was supplemented for osteoclast differentiation (*n* = 5 per group, ***P < 0.001). **P < 0.01, ***P < 0.001 (one-way ANOVA test followed by Dunn’s post-test for multiple comparisons, **a**, **b**)

To further illustrate the osteoclast-priming effects of Tyro3TK on CD14^+^CD16^−^ monocytes in RA patients, we performed osteoclast differentiation assay with or without Tyro3TK blockade. As shown in Fig. 5a, the co-culture of CD14^+^CD16^−^ monocytes isolated from RA patients with rhGas6 promoted TRAP-positive osteoclast formation, especially at the dose of 50 ng/ml. Strikingly, anti-Tyro3TK antibody significantly compromised this rhGas6-mediated exacerbation of osteoclast differentiation in a dose-dependent manner. At the dose of 200 ng/ml, anti-Tyro3TK antibody could almost abolish the formation of osteoclasts (Fig. 5b). Collectively, these results revealed the critical role of Tyro3TK in mediating CD14^+^CD16^−^ monocyte differentiation into osteoclasts.

## Discussion

In this study, we found that CD14^+^CD16^−^ monocytes were more potent in osteoclast differentiation in HC, the capacity of which was more powerful in RA patients. The expression of Tyro3TK on CD14^+^CD16^−^ monocytes were upregulated in RA, positively correlating with the clinical features of the patients. Moreover, upregulated Tyro3TK on CD14^+^CD16^−^ monocytes promotes their osteoclast differentiation in RA.

Peripheral blood monocytes played an essential role in secreting inflammatory factors, regulating innate immunity, and inducing osteoclast formation [28]. Monocyte heterogeneity has been recognized in humans for a long time. Based on phenotypic characteristics, human monocytes can be divided into CD14^+^CD16^+^ and CD14^+^CD16^−^ monocytes, and the CD14^+^CD16^+^ monocytes can be further divided into non-classical (CD14^+^CD16^++^) and intermediate (CD14^++^CD16^+^) monocytes [7]. In this study, we mainly focus on the role of CD14^+^CD16^+^ and CD14^+^CD16^−^ monocytes in osteoclast formation with Tyro3TK expression. Nevertheless, the different roles of non-classical and intermediate monocytes in osteoclastogenesis, as well as the involvement of Tyro3TK are of significance, which will be revealed in our future study. In addition, it should be noticed that there is also a specific subset of DCs derived from monocytes known to express CD14 but not CD16 (named Mo-DC) [29]. Our result showed that the frequencies of Mo-DC in CD14^+^CD16^−^ cells were ~ 1.54% (data not shown). This might induce minimal interference to the current results yet could not be excluded, and the role of Mo-DC in osteoclastogenesis deserves to be further studied.

CD14^+^CD16^+^ and CD14^+^CD16^−^ monocyte subsets might possess different functions in RA. Our previous study showed that CD14^+^CD16^+^ monocytes in patients with systemic lupus erythematosus showed inflammatory phenotype, with increased CD80, CD86, HLA-DR, and CX3CR1, which could promote Th17 response [30]. IL-17 is a pro-inflammatory cytokine mainly produced by CD4^+^ T cells and plays a critical role in RA synovitis [31]. Kotake et al. illustrated that the level of cytokine IL-17 was significantly increased in RA synovial fluid, and IL-17 could promote osteoclast differentiation from CD14^+^ monocytes [32]. CD14^+^CD16^+^ monocytes can also migrate to RA synovium and produce high levels of TNF-α, IL-6, and IL-1β. These cytokines could promote the production of cytokine IL-17, thus playing a critical role in synovial inflammation and osteoclasts formation [33–35]. Here, we showed that CD14^+^CD16^−^ monocytes were more prone to differentiate into osteoclasts than CD14^+^CD16^+^ monocytes in healthy controls. Moreover, the osteoclastic capacity of CD14^+^CD16^−^ monocytes was significantly enhanced in RA patients. Although with controversial, these results were consistent with most previous studies [10–12]. Therefore, we speculate that CD14^+^CD16^−^ monocytes are the main osteoclast precursors in RA, while CD14^+^CD16^+^ monocytes are more competent in producing pro-inflammatory cytokines. Detailed mechanistic studies are still needed to reveal the differential functions of these two monocyte subsets.

Tyro3TK was initially discovered as a therapeutic target in tumors [36]. Increasing studies have focused on their critical role in autoimmune diseases [37, 38]. Barth et al. demonstrated that Tyro3TK could express in monocytes [39]. As the ligand of Tyro3TK, Gas6 was evaluated in RA synovium tissue and fluid [19]. It can promote RA synovial hyperplasia, which is hallmarked by the abundant synovial fibroblasts and associated with bone destruction in RA [40]. Besides, Gas6-Tyro3TK interaction may play a critical osteoclast-priming role [21–24]. In this study, we showed that Tyro3TK on CD14^+^CD16^−^ monocytes of RA patients was significantly upregulated, which was associated with clinical features and disease activity. Furthermore, Gas6 can promote the osteoclasts formation of CD14^+^CD16^−^ monocytes, while disrupts Gas6-Tyro3TK interaction, the number of osteoclasts differentiated from CD14^+^CD16^−^ monocytes decreased significantly with a dose-dependent anti-Tyro3TK antibody. The study also extends our findings, demonstrating that Tyro3TK has a distinct role in regulating CD14^+^CD16^−^ monocyte osteoclastogenesis, suggesting that Tyro3TK might be a possible therapeutic target for RA bone destruction. Therefore, it is intriguing to propose that targeting Tyro3TK and CD14^+^CD16^−^ monocytes simultaneously may have a more apparent inhibitory effect on bone destruction in RA. However, the detailed signal mechanisms of Tyro3TK on CD14^+^CD16^−^ in RA need to be further studied.

## Conclusion

In summary, this study reveals that CD14^+^CD16^−^ monocytes are the main precursors of osteoclasts in RA. Moreover, upregulated Tyro3TK expression on these cells provides a pivotal role for osteoclastogenesis, which might serve as therapeutic targets for the persistent disease.

## Data Availability

The datasets used and/or analyzed during the present study are available from the corresponding author on reasonable request.

## Abbreviations

RA: Rheumatoid arthritis
OA: Osteoarthritis
HC: Healthy control
TRAP: Tartrate-resistant acid phosphatase
Gas6: Growth arrest-specific protein 6
ProS1: Protein S
RTKs: Receptor tyrosine kinases
ACR: American College of Rheumatology
EULAR: European League Against Rheumatism
SJC: Swollen joint count
TJC: Tender joint count
WBC: White blood cells
RBC: Red blood cells
Hb: Hemoglobin
PLT: Platelets
IgA/G/M: Immunoglobulin
A/G/M: Anti-CCP antibody, anti-cyclic citrullinated peptide antibody
ESR: Erythrocyte sedimentation rate
CRP: C-reactive protein
PBMCs: Peripheral blood mononuclear cells
PBS: Phosphate-buffered saline
FMO: Fluorochrome-matched controls
MFI: Mean fluorescence intensity
α-MEM: α-Minimum Essential Medium
M-CSF: Macrophage colony-stimulating factor
RANKL: Nuclear factor-κB ligand
